# Pilot study of a Spanish language measure of financial toxicity in underserved Hispanic cancer patients with low English proficiency

**DOI:** 10.3389/fpsyg.2023.1188783

**Published:** 2023-07-10

**Authors:** Julia J. Shi, Gwendolyn J. McGinnis, Susan K. Peterson, Nicolette Taku, Ying-Shiuan Chen, Robert K. Yu, Chi-Fang Wu, Tito R. Mendoza, Sanjay S. Shete, Hilary Ma, Robert J. Volk, Sharon H. Giordano, Ya-Chen T. Shih, Diem-Khanh Nguyen, Kelsey W. Kaiser, Grace L. Smith

**Affiliations:** ^1^Department of Radiation Oncology, The University of Texas MD Anderson Cancer Center, Houston, TX, United States; ^2^Department of Behavioral Science, The University of Texas MD Anderson Cancer Center, Houston, TX, United States; ^3^Department of Biostatistics, The University of Texas MD Anderson Cancer Center, Houston, TX, United States; ^4^Department of Health Services Research, The University of Texas MD Anderson Cancer Center, Houston, TX, United States; ^5^Department of Symptom Research, The University of Texas MD Anderson Cancer Center, Houston, TX, United States; ^6^Department of General Oncology, The University of Texas MD Anderson Cancer Center, Houston, TX, United States; ^7^University of California Riverside School of Medicine, Riverside, CA, United States; ^8^Department of Gastrointestinal Radiation Oncology, The University of Texas MD Anderson Cancer Center, Houston, TX, United States

**Keywords:** Spanish, financial toxicity, English proficiency, Hispanic, underserved, ENRICh, cancer, health insurance

## Abstract

**Background:**

Financial toxicity (FT) reflects multi-dimensional personal economic hardships borne by cancer patients. It is unknown whether measures of FT—to date derived largely from English-speakers—adequately capture economic experiences and financial hardships of medically underserved low English proficiency US Hispanic cancer patients. We piloted a Spanish language FT instrument in this population.

**Methods:**

We piloted a Spanish version of the Economic Strain and Resilience in Cancer (ENRICh) FT measure using qualitative cognitive interviews and surveys in un-/under-insured or medically underserved, low English proficiency, Spanish-speaking Hispanics (UN-Spanish, *n* = 23) receiving ambulatory oncology care at a public healthcare safety net hospital in the Houston metropolitan area. Exploratory analyses compared ENRICh FT scores amongst the UN-Spanish group to: (1) un-/under-insured English-speaking Hispanics (UN-English, *n* = 23) from the same public facility and (2) insured English-speaking Hispanics (INS-English, *n* = 31) from an academic comprehensive cancer center. Multivariable logistic models compared the outcome of severe FT (score > 6).

**Results:**

UN-Spanish Hispanic participants reported high acceptability of the instrument (only 0% responded that the instrument was “very difficult to answer” and 4% that it was “very difficult to understand the questions”; 8% responded that it was “very difficult to remember resources used” and 8% that it was “very difficult to remember the burdens experienced”; and 4% responded that it was “very uncomfortable to respond”). Internal consistency of the FT measure was high (Cronbach’s *α* = 0.906). In qualitative responses, UN-Spanish Hispanics frequently identified a total lack of credit, savings, or income and food insecurity as aspects contributing to FT. UN-Spanish and UN-English Hispanic patients were younger, had lower education and income, resided in socioeconomically deprived neighborhoods and had more advanced cancer vs. INS-English Hispanics. There was a higher likelihood of severe FT in UN-Spanish (OR = 2.73, 95% CI 0.77–9.70; *p* = 0.12) and UN-English (OR = 4.13, 95% CI 1.13–15.12; *p* = 0.03) vs. INS-English Hispanics. A higher likelihood of severely depleted FT coping resources occurred in UN-Spanish (OR = 4.00, 95% CI 1.07–14.92; *p* = 0.04) and UN-English (OR = 5.73, 95% CI 1.49–22.1; *p* = 0.01) vs. INS-English. The likelihood of FT did not differ between UN-Spanish and UN-English in both models (*p* = 0.59 and *p* = 0.62 respectively).

**Conclusion:**

In medically underserved, uninsured Hispanic patients with cancer, comprehensive Spanish-language FT assessment in low English proficiency participants was feasible, acceptable, and internally consistent. Future studies employing tailored FT assessment and intervention should encompass the key privations and hardships in this population.

## 1. Introduction

Financial toxicity (FT) reflects the personal economic burden borne by individuals with cancer (Zafar et al., 2013). FT results from the direct and indirect costs of treatment and disease, and it manifests in a variety of ways such as the accrual of medical debt, non-adherence to treatment due to cost, and development of psychological distress related to financial concerns. In prior studies, as many as half of individuals with cancer in the US were found to experience FT during treatment or survivorship (Altice et al., 2017). Prior studies have also suggested that racial and ethnic minorities have especially high prevalence of FT, attributed to greater socioeconomic vulnerability from lower income and higher rates of un- or under-insurance (Bernard et al., 2011; Kent et al., 2013; Nipp et al., 2016; Kaul et al., 2017; Zheng et al., 2017). In the US, evidence suggests that the Hispanic population overall has lower population-level educational attainment, household income, and English language proficiency as well as the highest uninsured rate of any racial or ethnic group (Office of Minority Health, 2022). These elements have been shown to place Hispanic populations at especially high risk for decreased healthcare access and more advanced cancer at diagnosis (Chebli et al., 2020). Non-citizen status is an additional factor that can potentiate these challenges (Ashing-Giwa et al., 2006; Buki et al., 2008; Simon et al., 2013; Azzani et al., 2015; Lentz et al., 2019). As a result, Hispanic patients with cancer have substantial risks for developing FT.

However, conflicting evidence exists on the severity and spectrum of FT in US Hispanics. For example, 42% of Hispanics with a cancer history in the 2010 National Health Interview Survey reported a negative financial impact compared with 33% of non-Hispanic whites (Ashing-Giwa et al., 2006). In contrast, a recent analysis of the 2012, 2014, and 2017 Health Information National Trends Surveys (HINTS) did not find a significant difference between Hispanics and non-Hispanic white respondents reporting that they were hurt financially due to cancer (Panzone et al., 2021). Comprehensive measurement and assessment of FT in Hispanic cancer patients and survivors are therefore still needed to improve nuanced understanding of severity, sources, dimensions, and mitigators of FT in this population.

In addition, given the large population of US Hispanics with low English proficiency and the relationship between low English proficiency and quality of care, there is a need for Spanish language tools to measure FT that adequately encompass and represent the aspects of financial hardship that this population experiences. Advancing assessment of FT in US Hispanic patients and survivors with cancer will promote early identification, inform tailored interventions, and enhance delivery of community-partnered health resources for high-risk individuals in this population.

To advance the assessment and understanding of FT in low English proficiency Hispanic cancer patients, we developed and piloted a Spanish language version of the previously validated Economic Strain and Resilience in Cancer (ENRICh) FT measure (Smith et al., 2021; Xu et al., 2022). The English version of the ENRICh measure has been psychometrically validated and has been used in prior studies to identify risk factors and outcomes in cancer patients with FT. In addition, the English version of the instrument has been useful for measuring the severity of subdomains of FT, including material burdens, coping resource depletion, and the psychological burden of FT (Maldonado et al., 2021; Corrigan et al., 2022).

The primary objective of this pilot study was to evaluate the acceptability and appropriateness of the Spanish language instrument for assessing FT in a pilot sample of un-/under-insured or medically underserved low English proficiency Hispanic individuals receiving ambulatory oncology care from a public medical safety net hospital. This hospital is in the Houston metropolitan area, Texas, where persons of Hispanic ethnicity comprise approximately 45% of the population. The study’s secondary objective was to conduct exploratory analyses of the impact of insurance status and English language proficiency on FT outcomes. To address this objective, we conducted exploratory comparisons of FT outcomes reported in the pilot sample of un-/under-insured and underserved low English proficiency Hispanics (UN-Spanish) with the FT outcomes of two other groups with a historical comparison with: (1) un-/under-insured and medically underserved English-speaking Hispanics (UN-English) and (2) insured English-speaking (INS-English) Hispanics drawn from the parent study of the English version ENRICh FT instrument psychometric validation analysis (Smith et al., 2021).

## 2. Materials and methods

This study was approved by the University of Texas M. D. Anderson Cancer Center Institutional Review Board. All participants provided informed consent or waiver of signed consent per protocol.

### 2.1. Study population

#### 2.1.1. Spanish language sample (UN-Spanish)

Individuals were eligible for survey and cognitive qualitative interviews if they were aged ≥18 years, had confirmed diagnosis of cancer, indicated in their medical record that they required Spanish language interpretation for care, and were receiving ambulatory oncology care (active cancer treatment or follow-up/surveillance care) at the Lyndon B Johnson Hospital Oncology Clinic (LBJ) in Houston, Texas. LBJ is a facility in the county public health system which provides care for medically underserved and un- or under-insured patients with a household income of <150% of the Federal Poverty Level in partnership with the county-based healthcare safety net. This facility provides financial assistance through sliding scale out-of-pocket medical charges based on income level (Patient Eligibility, 2022).

Participants in this study were selected as a purposive sample of patients who presented for care at the clinic during 12 select clinic dates (based on research staff availability) between November 2020 and May of 2021 who were approached for study participation. The individual’s need for Spanish language interpretation indicated in the medical record was confirmed in person by the research staff prior to study enrollment. Of the 27 patients who were approached for participation, four refused, making a total of *n* = 23 in the UN-Spanish Hispanic sample. As described, use of the “Spanish-speaking” category label (UN-Spanish) reflects low English language proficiency.

#### 2.1.2. Comparison sample (UN-English and INS-English)

The comparison English-speaking Hispanic samples were derived from the parent survey cohort assembled for the psychometric validation analysis in the Economic Strain and Resilience in Cancer study (ENRICh) and the short form validation (Smith et al., 2018, 2021; Xu et al., 2022). Eligibility criteria for the parent cohort were identical, except all participants were required to be able to read and complete the survey in English. English language preference in these participants was also confirmed at the time of study enrollment. Eligible patients received ambulatory oncology care between March and September 2019 at LBJ or the University of Texas M.D. Anderson Cancer Center (MDA), an academic National Cancer Institute (NCI) designated comprehensive cancer hospital. Among enrollees from the parent ENRICh survey (*N* = 312, a response rate of 63.5% from 491 invited), all Hispanic respondents (*n* = 31 from MDA and *n* = 23 from LBJ) were included in the present analysis.

#### 2.1.3. Definition of patient comparison groups

A total of three comparison groups were defined for this analysis: (1) un-/under-insured and underserved low English proficiency Hispanics (UN-Spanish) enrolled from the public clinic (*n* = 23); (2) un-/under-insured and underserved English-speaking Hispanics (UN-English) from the public clinic (*n* = 23); and (3) insured English-speaking Hispanics (INS-English) from the academic comprehensive care center (*n* = 31).

### 2.2. Development of the Spanish-language version of ENRICh

The Economic Strain and Resilience in Cancer (ENRICh) instrument is a 15-item measure of patient-reported financial toxicity (FT) that is psychometrically validated among English-speaking survey respondents (Smith et al., 2021). The instrument scores overall FT. In addition, it scores FT in three subdomains: (1) material hardship such as out-of-pocket medical costs, spent savings, accumulated credit card or other debt, and lost income; (2) depletion of coping resources such as employment benefits, professional assistance from formal resources (e.g., professional organizations, charities), and informal support (e.g., from family and friends); and (3) related psychological burdens such as stress related costs or financial hardship. Respondents rate each item and the final scores, including overall score and each subdomain score, range from 0 to 10 (with higher scores indicating more severe burden).

#### 2.2.1. Spanish instrument translation and assessment

For this pilot, the ENRICh instrument was translated into Spanish through iterative forward translation followed by backward translation harmonized for Latin American Spanish (Supplementary Table S1). To assess acceptability of the Spanish-language instrument, respondents participated in a cognitive debriefing and qualitative interviews as guided by the COnsolidated criteria for REporting Qualitative research (CORE-Q) criteria. Interviews and instrument administration were conducted in Spanish by bilingual members of the study team. Sessions were audio-recorded and transcribed verbatim and analyzed in English using a deductive approach based on the existing conceptual model of FT delineating major subdomains of FT: material, psychological, and behavioral (Altice et al., 2017; Tucker-Seeley and Thorpe, 2019). Two independent coders (GM and GS) analyzed the data.

Participants first completed the Spanish version ENRICh instrument and then were asked to rate items on a 6-item questionnaire regarding aspects of usability, relevance, comprehension, and ease of response to instrument items (with a score of 0 representing very usable, relevant, easy to understand etc. and score of 8–10 categorized to represent very difficult to use, not relevant, very difficult to understand etc. Therefore, lower scores on these scales represent higher acceptability).

Finally, participants were also asked to reflect on their qualitative understanding of the constructs and concepts in each item using their own words. Each item was followed by open-ended qualitative interview probes by the interviewer to determine whether there were additional aspects of economic burden and financial hardship. If literacy was a barrier or the participant expressed such a preference, the interviewer read aloud both the questions and answer options for the participant. Quantitative score responses were summarized, and qualitative responses were coded for representative quotes on themes and subdimensions of FT.

### 2.3. Financial toxicity instrument scoring

An overall FT score and material FT, coping FT, and psychological FT domain subscores were calculated as an arithmetic average of item scores (re-weighted for missing items based on the total number of items completed) as previously reported in the original English psychometric validation analysis (Smith et al., 2021). In the original scoring, surveys with more than half of the items with missing responses were considered invalid. In this study, no respondent had more than half of survey items as missing responses, so scores for all respondents were included in analysis.

FT scores were further analyzed as continuous or dichotomous outcomes in multivariate models. The dichotomous cut point was defined as severe FT, indicated by a score >6. This approach for dichotomization was adapted based on prior findings demonstrating that a cut point score at 5 defining severe FT predicted the adverse outcomes of accumulation of medical debt and non-adherence to medical care (Maldonado et al., 2021). Because of the shift in distribution of scores toward higher scores with more severe FT in the present study sample (attributable to the high percentage of underserved, uninsured individuals), the cut point was defined at 6 for this analysis.

### 2.4. Ethnicity and other covariates

*Hispanic ethnicity and patient race*. Data on self-identified ethnicity and race were abstracted from each respondent’s medical record. These fields are pre-determined menu options which patients select as a component of routine registration for care at both facilities. In the MDA medical record, respondents may select “Hispanic or Latino/a” or “Not Hispanic or Latino/a” for ethnicity. In the LBJ medical record, respondents may select the same options, with another submenu option if “Hispanic or Latino/a” was selected to further categorize ethnicity as “Mexican, Mexican American, or Chicano/a” vs. “Other Hispanic, Latino/a, or Spanish origin.” At LBJ, “Hispanic/Latino/a” is also offered as an option for race, but it is not available as an option for race at MDA. Other race categories of respondents in this sample were “Black or African-American” and “American Indian,” based on respondents’ category selection for race in the medical record.

#### 2.4.1. Sociodemographic and clinical covariates

Age at survey, home address, gender, education, work status, marital status, race, ethnicity, primary cancer disease site (e.g., breast, lung, prostate, etc.), and cancer stage at diagnosis (categorized as local vs. regional or advanced or metastatic, adopted from the Surveillance Epidemiology and End Results summary stage framework) were also collected. Respondents were surveyed for total household income, health insurance status [including public (e.g., Medicaid, Medicare, other state programs), private (employer-purchased, self-purchased), or uninsured], highest attained level of education, and current work status. Home address zip codes were linked to Federal Information Processing System (FIPs) codes to calculate Area Deprivation Index (ADI) scores. The ADI scores the individual’s neighborhood-based socioeconomic deprivation level and has been previously found to be associated with healthcare outcomes (Ludwig et al., 2011; Hu et al., 2018; Neighborhood Atlas – Home, 2022). ADI scores range from 1 to 100 (least to greatest severity of neighborhood deprivation, respectively) normalized based on national percentile ranking across neighborhoods in the US. Categories used in univariate and multivariable analyses were based on variables’ distributions.

### 2.5. Statistical analyses

Internal consistency of item scoring for the overall FT measure was tested for each patient group (UN-Spanish, UN-English, INS-English) using Cronbach’s *α*. Univariate associations between covariates and patient groups, and overall FT score were tested using the Wilcoxon Rank Sum Test and Fisher’s Exact Test. Multivariable logistic models were tested to identify the adjusted associations for patient groups and odds of severe overall FT and subdomain FT (scores ≥6). Covariates were considered for retention in the models if they demonstrated univariate associations with *p* ≤ 0.05. Final parsimonious models were derived based on retaining important covariates identified in prior studies of FT (age and sex) (Smith et al., 2021). Insurance status, ADI, chemotherapy, and advanced/metastatic cancer were tested but excluded as final covariates due to non-significance, collinearity with the main independent variable of interest (patient group), or the models based on Akaike information criterion (AIC) lacking goodness of fit. Models were performed on the entire cohort with the INS-English group as the referent category compared with UN-English and with UN-Spanish groups. Secondarily, models were performed on the subset only of un- or underinsured patients to directly compare the UN-English vs. UN-Spanish groups to explore the effect of English language proficiency on FT outcomes. Analyses were conducted using SAS Enterprise Guide version 7.11 (Cary, NC). Statistical tests were two-sided with a *p* value ≤0.05 considered statistically significant.

## 3. Results

Among all participants (*N* = 77), the mean age was 50.2 (SD 14.4) years with a variety of cancer types: breast 39.0% (*n* = 30), gastrointestinal 20.8% (*n* = 16), hematologic 9.1% (*n* = 7), lung 9.1% (*n* = 7), genitourinary 7.8% (*n* = 6), soft tissue 6.5% (*n* = 5), and other 7.8% (*n* = 6). Among the patients who specified the subcategory of Hispanic ethnicity, 39% (18 of 46) specified “Mexican, Mexican American, or Chicano/a.” One patient specified American Indian race and two patients specified Black or African-American race.

UN-Spanish and UN-English Hispanics tended to be younger and have lower education and lower income than INS-English Hispanics. Home neighborhood deprivation indicated by mean ADI score differed between groups, with Hispanic patients from the UN-Spanish [75.1, standard deviation (SD) 16.6] and UN-English (65.0, SD 23.8) groups living in more deprived neighborhoods vs. INS-English Hispanics (54.9, SD 25.8) (*p* = 0.03). All INS-English Hispanics had health insurance. Other detailed characteristics compared between groups are shown in Table 1.

**Table 1:** Participant characteristics and comparisons between insured English-speaking (INS-English), un- and under-insured English-speaking, and un- and under-insured low English proficiency Hispanic individuals with cancer.

Patient characteristics	INS-English	UN-English	UN-Spanish	*p*-value
	(*n* of 31, %)	(*n* of 23, %)	(*n* of 23, %)	
Age, Mean, standard deviation (SD)	55.6, 14.0	44.1, 13.4	49.1, 13.8	**0.023**
Gender % (*n*)				0.37
Male	9 (29.0%)	11 (47.8%)	7 (30.4%)	
Female	22 (71.0%)	12 (52.2%)	16 (69.6%)	
Neighborhood Area Deprivation Index Score, Mean, SD	54.9, 25.8	65.0, 23.8	75.1, 16.6	**0.03**
Currently Working for Pay				**0.001**
Yes	15 (48.39%)	3 (13.04%)	2 (8.70%)	
No	15 (48.39%)	20 (86.96%)	21 (91.30%)	
Income				**<0.001**
<$10,000	2 (6.45%)	10 (43.48%)	6 (26.09%)	
$10,000–$34,999	4 (12.90%)	12 (52.17%)	6 (26.09%)	
$35,000–$49,999	3 (9.68%)	1 (4.35%)	0 (0%)	
$50,000–$99,999	13 (41.94%)	0 (0%)	0 (0%)	
>$100,000	8 (25.81%)	0 (0%)	0 (0%)	
No Response	1 (3.3%)	0 (0%)	11 (47.8%)	
Education				**<0.001**
Less than High School, High School, or GED	10 (32.26%)	15 (65.22%)	14 (60.87%)	
Some College, Associate Degree, or Trade Certification	14 (45.16%)	8 (34.78%)	0 (0%)	
College, Graduate, or Advanced Degree	7 (22.58%)	0 (0%)	1 (4.35%)	
No Response	0 (0%)	0 (0%)	8 (34.78%)	
Marital Status				0.41
Married or Living as Married	18 (58.06%)	9 (39.13%)	11 (47.83%)	
Other	13 (41.94%)	14 (60.87%)	12 (52.17%)	
Insurance				**<0.001**
Private (Employer or Purchased)	28 (90.32%)	1 (4.35%)	0 (0%)	
Medicaid or Other State	0 (0%)	6 (26.09%)	2 (8.70%)	
Medicare	3 (9.68%)	1 (4.35%)	1 (4.35%)	
No Insurance	0 (0%)	15 (65.22%)	20 (86.96%)	
Received Chemotherapy				0.10
Yes	23 (74.2%)	20 (87.0%)	22 (95.7%)	
No	8 (25.8%)	3 (13.0%)	1 (4.4%)	
Advanced or Metastatic Cancer				**0.001**
Yes	16 (51.6%)	14 (60.9%)	22 (95.7%)	
No	15 (48.4%)	9 (39.1%)	1 (4.4%)	
Disease Site				
Breast	16 (51.6%)	8 (34.8%)	6 (26.1%)	0.34
Gastrointestinal	4 (12.9%)	5 (21.7%)	7 (30.4%)	
Other*	11 (35.5%)	10 (43.5%)	10 (43.5%)	
Median Interval from Diagnosis in days (interquartile range)	297 (82, 575)	270 (99, 546)	860 (173, 1,687)	**0.006**

*Other disease sites include: central nervous system, head and neck, hematologic (leukemia, lymphoma, myeloma), lung, genitourinary, gynecologic, sarcoma and other soft tissue, and thymic malignancies. Bold values indicate *p* < 0.05.

### 3.1. Spanish language FT measure cognitive debriefing and acceptability

Among the UN-Spanish Hispanic group, there was high acceptability of the instrument. Most respondents found it easy to answer the questions (median 0, IQR 0–1 with a lower score representing greater ease in responding; 0% very difficult), easy to understand the questions (median 0, IQR 0–3 with a lower score representing greater ease in understanding; 4% score of very difficult), felt comfortable responding to the questions (median 0, IQR 0–1 with a lower score representing greater comfort in responding; 4% very uncomfortable), felt it was easy to remember the resources that were offered in the last month (median 0, IQR 0–1 with a lower score representing greater ease in recall; 8% very difficult), and felt it was easy to remember the financial burdens of the last month (median 0, IQR 0–2; 8% very difficult). Some respondents found questions repetitive (median 0, IQR 0–3 with a lower score representing greater comfort with the level of repetition; 17% very repetitive). Only two respondents found the 0 to 10 scale difficult to understand and expressed that they would prefer binary options only (yes or no) for items.

Regarding the cognitive interviews, UN-Spanish Hispanic respondents still identified frequent material hardships attributed to a lack of savings, medical bills, insurance coverage difficulties, and income losses (Table 2). These respondents identified that a complete lack of savings, credit, or income was an underlying rationale for choosing a score of “0” in material FT items, but there was a conflict in scoring, with “0” being a response that represented a complete lack of the resource vs. “10” being a response that represented the severe hardship from lacking that resource. Individual item scoring for material FT items demonstrated that respondents frequently scored “0” (Table 3). For example, the frequency of a “0” score for the following items were: cancer or cancer treatment impacted “money in savings” (33.3%), “spending from savings” (36.4%), and “credit card use” (75.0%). Therefore, qualitatively, respondents expressed that an option of “I do not have savings/credit cards/income” could be added for these items to better tailor to their circumstances.

**Table 2:** Qualitative responses on aspects of material, coping, and psychological financial toxicity during cognitive interviewing and linguistic validation, translated from Spanish.

Hardships
**Depletion/lack of pre-existing assets**
“Without working, the finances are gone, and you depend on people’s help.”
“I cannot provide like—like what we need at home. Let’s say, if we need to buy something additional for our home, or if I need emergency money, I do not have it. I cannot do that because I paying for everything else.”
“I don’t have any savings from the time I used to work.”
**Lack of credit and credit card access**
“We do not use credit cards.”
“I don’t have credit cards.”
“I don’t have a credit card.”
**Food insecurity**
“Once every fifteen days, thank God, a church provides us with food.”
“As I have paid for gas, to come here—I have paid for gas, I have—well, I’ve been hungry and not wanting to buy any little thing, and that’s where all the money is going.”
**Transportation difficulties**
“Well, I don’t drive. I don’t have the—I drive, but I don’t have a car. I have to depend on someone else to get me around.”
“A lot of people do struggle, for instance, to find transportation to come to the hospital. Or many people do not have the ten dollars or the six—eight dollars that they charge you for parking.”
“Sometimes I don’t have anybody who can give me a ride to the hospital, and I have to spend my money to pay for taxis or transportation.”
**Immigration difficulties**
“If you don’t have a backup, an insurance, which you cannot have as Hispanic and immigrant here, undocumented, unfortunately—I mean, it gets very difficult.”

Burdens
**Medical bills**
“When I was first diagnosed, I did not have insurance, as I told you, and what they needed was urgent because it was a stage four cancer with metastasis, and I needed to get the treatment immediately. And so just in order to start getting treatment, I needed an average of 5,000 dollars, which I didn’t have.”
“Yes, because before getting sick, I never had medical expenses, and now I have a pending bill of 4,000 dollars.”
**Lack of insurance**
“At the beginning, it was a hard blow, and it affected me a lot because I didn’t have medical insurance; I didn’t have anywhere—nothing to help me cover the expenses of a disease like this one.”
**Income loss**
“The cancer caused me to have bone problems, and I fractured my back. Imagine how hard it was to work. I fractured my back twice, three vertebrae, and the last time I fractured two vertebrae, and I cannot work. It is even harder because it is a construction job. And that affects me.”
“When this began, I had to stop working, and therefore, I stopped having money, you know?”
“I went from earning, let’s say, 5,000 dollars per month, to earning—to maybe earning 100 dollars per day, in the—when I started the treatment—the disease. So there were times when I could not do anything.”
“When I got sick, I had to quit my job.”
**Impairment in caregiving responsibilities**
“I can no longer take care of her. It did affect my ability to take care of my child.”
“Always—all the time, since I started this treatment—this case—the responsibility has fallen more on her, on my wife, because when it was the two of us—that is, 100 percent—it was less of a burden on both of us.”
“It is supposed to be me who should be taking care of the cousins when my aunt is not there, but I struggle a lot.”

Mitigating factors
**Social network support resources**
“I borrowed money from relatives, friends in order to start with the treatment because I didn’t have any type of insurance.”
“Well, they helped me with my groceries, people from the church. They gave me food, thank God.”
“Like churches—they help you with a percentage.”
“Right now, I cannot work. Sometimes I have to pay for the rent or for the food to my aunt. My dad got in a very bad financial state when he had to pay for the chemo, so I’m nervous that they are going to—to lose my insurance or something like that.”
**Formal assistance resources**
“Thank God my wife was able to find—well, she applied to get the gold card (public clinic financial assistance program), and I was lucky that they gave it to me, right?”
“The gold card (public clinic financial assistance program), yes. I get that help.”

**Table 3:** Median scores and frequency of response of score “0” for individual items, subscores, and overall scores for financial toxicity (FT) compared between groups.

Item	UN-Spanish				UN-English					INS-English				*p* value 3 groups
	*n* of 23	Median	IQR	% “0”	*n* of 23	Median	IQR	% “0”	*p* value 2 groups	*n* of 31	Median	IQR	% “0”	
**Material FT subdomain**														
Money in savings	21	3.0	0.0–9.0	33.3%	23	10.0	7.0–10.0	8.7%	**0.01**	28	5.0	2.0–10.0	17.9%	**0.01**
Other money owed	23	0.0	0.0–7.0	52.2%	22	8.5	0.0–10.0	27.3%	**0.03**	31	5.0	0.0–8.0	35.5%	0.06
Medical spending	23	2.0	0.0–8.0	34.8%	21	7.0	2.0–9.0	14.3%	0.17	31	6.0	2.0–9.0	16.1%	0.25
Spending household income	23	5.0	2.0–9.0	13.0%	23	5.0	2.0–10.0	17.4%	0.59	31	6.0	2.0–9.0	16.1%	0.84
Spending from savings	22	2.0	0.0–8.0	36.4%	23	6.0	0.0–10.0	26.1%	0.21	31	3.0	0.0–6.0	29.0%	0.31
Credit card use	20	0.0	0.0–2.0	75.0%	23	0.0	0.0–8.0	60.9%	0.35	31	3.0	0.0–6.0	38.7%	0.14
**Material FT subscore**	23	3.3	0.8–6.7	13.0%	23	5.8	3.3–8.3	0.0%	**0.04**	31	4.3	2.4–7.3	6.5%	0.10
**Coping FT subdomain**														
Ability to pay all bills	23	5.0	0.0–8.0	26.1%	23	10.0	0.0–10.0	26.1%	0.11	31	2.0	0.0–7.0	38.7%	**0.01**
Ability to pay for food	22	4.0	0.0–9.0	27.3%	23	8.0	2.0–10.0	13.0%	0.22	30	0.0	0.0–1.0	66.7%	**<0.001**
Ability to work usual number of hours at job	22	10.0	9.0–10.0	18.2%	23	10.0	9.0–10.0	17.4%	0.92	31	0.0	0.0–8.0	58.1%	**<0.001**
Ability to contribute to typical household responsibilities	23	6.0	3.0–10.0	17.4%	23	9.0	6.0–10.0	8.7%	0.21	29	3.0	0.0–8.0	37.9%	**0.01**
Assistance with managing medical bills	22	3.0	0.0–10.0	45.5%	23	4.0	0.0–10.0	39.1%	0.81	30	0.0	0.0–4.0	73.3%	**0.04**
Assistance with typical responsibilities	23	8.0	1.0–10.0	21.7%	23	9.0	5.0–10.0	17.4%	0.6	30	2.5	0.0–8.0	40.0%	**0.04**
Assistance with care for dependents	23	5.0	0.0–10.0	39.1%	23	5.0	0.0–10.0	34.8%	0.87	31	0.0	0.0–5.0	54.8%	0.07
Assistance from community	23	0.0	0.0–6.0	60.9%	23	0.0	0.0–9.0	56.5%	0.73	30	0.0	0.0–0.0	76.7%	0.16
**Coping FT sub score**	23	5.0	2.5–8.0	4.3%	23	6.3	3.0–8.4	0.0%	0.31	31	2.4	0.6–4.6	9.7%	**<0.001**
**Psychological FT subdomain**														
Stress level about finances	23	7.0	1.0–10.0	21.7%	21	10.0	7.0–10.0	0.0%	0.05	30	7.0	2.0–10.0	10.0%	**0.05**
**Psychologic FT subscore**	23	7.0	1.0–10.0	21.7%	21	10.0	7.0–10.0	0.0%	0.05	30	7.0	2.0–10.0	10.0%	**0.05**
**Overall FT score**	23	5.4	1.5–7.1	4.3%	23	6.0	3.4–8.3	0.0%	0.12	31	3.2	1.9–5.3	6.5%	**0.01**

UN, un-/under-insured and underserved; INS, insured; IQR, interquartile range; FT, financial toxicity. Bold values indicate *p* < 0.05.

In qualitative interviews, respondents also identified additional specific dimensions of cancer-related FT, including the impact of undocumented immigration status and lack of basic resources such as food, housing, and transportation. Respondents further identified potential factors that mitigated FT, including support resources through church and family. The financial assistance program offered through the public safety-net hospital was another key mitigating factor (Table 2).

### 3.2. Financial toxicity scores: exploratory comparison of severity and dimensions by patient groups

Cronbach’s *α* values demonstrated high internal consistency for measuring the underlying construct of overall FT in each group: UN-Spanish = 0.906, UN-English = 0.904, and INS-English = 0.906. The median scores for overall FT were similar for UN-Spanish (5.4, IQR 1.5–7.1) vs. UN-English (6.0, IQR 3.4–8.3) groups (*p* = 0.12). However, the INS-English group had significantly less severe overall FT, with a median score of 3.2 (IQR 1.9–5.3) (*p* = 0.01) (Table 3).

The subdomain scores for material FT, coping FT, and psychological FT are found in Table 3. The coping FT domain demonstrated the most substantial differences in median scores for UN-Spanish (5.0, IQR 2.5–8.0) and UN-English (6.3, IQR 3.0–8.4) Hispanics vs. INS-English (2.4, IQR 0.6–6.4) (*p* < 0.001). Other covariate correlates of FT are shown in Supplementary Table S2.

In multivariable models, compared with INS-English Hispanics, the likelihood of severe overall FT (score > 6) was significantly increased for UN-English Hispanics [Odds Ratio (OR) = 4.13, 95% Confidence Interval (CI) 1.13–15.12; *p* = 0.03] and increased with borderline statistical significance for UN-Spanish Hispanics (OR = 2.73, 95% CI 0.77–9.70; *p* = 0.12). Compared with INS-English Hispanics, there was a significantly higher likelihood of severe coping FT for both UN-English (OR = 5.73, 95% CI 1.49–22.1; *p* = 0.01) and UN-Spanish Hispanics (OR = 4.00, 95% CI 1.07–14.92; *p* = 0.04) (Table 4). In the subset analysis including only the un-/under-insured groups, there were no significant differences detected for UN-Spanish Hispanics vs. UN-English Hispanics for overall FT (*p* = 0.59), material FT (*p* = 0.32), coping FT (*p* = 0.62), or psychological FT (*p* = 0.44). (Table 2).

**Table 4:** Multivariable logistic models of severe (score > 6) overall, material, coping, and psychological financial toxicity (FT), for all patients (*N* = 77) and the subset of un-/under-insured and underserved patients (*N* = 46).

All Patients (*N* = 77)												
Covariate	Overall FT			Material FT			Coping FT			Psychological FT		
	OR	95% CI	*p*	OR	95% CI	*p*	OR	95% CI	*p*	OR	95% CI	*p*
**Group**												
INS-English	1.00	–	–	1.00	–	–	1.00	–	–	1.00	–	–
UN-English	4.13	1.13–15.12	**0.03**	2.13	0.6–7.5	0.24	5.73	1.49–22.1	**0.01**	1.75	0.52–5.91	0.37
UN-Spanish	2.73	0.77–9.70	0.12	1.06	0.3–3.82	0.92	4.00	1.07–14.92	**0.04**	1.08	0.35–3.31	0.89
**Female**	0.47	0.17–1.31	0.15	0.42	0.15–1.18	1	0.40	0.14–1.17	0.09	0.53	0.19–1.46	0.22
**Age (years)**	1.00	0.96–1.04	0.98	1.00	0.97–1.05	0.66	0.99	0.96–1.03	0.75	1.00	0.97–1.04	0.86

Un-/under-insured and underserved Patient Subset (*N* = 46)												
Covariate	Overall FT			Material FT			Coping FT			Psychological FT		
	OR	95% CI	*p*	OR	95% CI	*p*	OR	95% CI	*p*	OR	95% CI	*p*
**Group**												
UN-English	1.00	–	–	1.00	–	–	1.00	–	–	1.00	–	–
UN-Spanish	0.71	0.21–2.44	0.59	0.52	0.14–1.91	0.32	0.74	0.22–2.52	0.62	0.60	0.17–2.18	0.44
**Female**	0.41	0.11–1.46	0.17	0.36	0.09–1.34	0.13	0.38	0.11–1.38	0.14	0.30	0.07–1.25	0.10
**Age (years)**	1.00	0.94–1.04	0.61	1.00	0.96–1.06	0.87	0.98	0.94–1.03	0.5	1.00	0.97–1.07	0.40

FT, financial toxicity; OR, odds ratio; CI, confidence interval; INS, insured; UN, un-/under-insured and underserved. Bold values indicate *p* < 0.05.

## 4. Discussion

FT is a critical source of financial anxiety and disparities in care delivery and health outcomes in cancer patients and survivors. Though early available data support that, overall, minority populations in the US have especially high prevalence of FT, comprehensive measurement of FT among Hispanics with cancer in the US remains lacking. The need for measuring FT is especially true for Hispanics who face serious socioeconomic barriers in order to advance FT screening and intervention in high-risk individuals. Our pilot study demonstrated the initial feasibility, acceptability, and internal consistency of a Spanish-language ENRICh measure to assess FT in low English proficiency Hispanic cancer patients using a translated version of a multi-dimensional validated tool for scoring FT (Smith et al., 2021). Our pilot focused on assessing a medically underserved Hispanic population with low English proficiency, un- or under-insurance, and high poverty level receiving care from a public safety net clinic. The low English proficiency Hispanic respondents in our study lived in highly disadvantaged neighborhoods in the larger Houston metropolitan area, with a mean ADI score representing the highest quartile of socioeconomic deprivation relative to neighborhoods across the US. The low English proficiency respondents in our study demonstrated low access to economic and health resources when compared with a control group of insured English-speaking Hispanics drawn from the same large metropolitan area.

The socioeconomic barriers and healthcare burdens observed in this low English language proficiency group in our study align with prior findings in Hispanic cancer survivors (Blinder et al., 2012; Jagsi et al., 2014; Lee and Salloum, 2016). Bilingualism among Hispanics in prior studies is associated with higher income (Katz et al., 2017), while low English language proficiency is associated with more recent immigration and socioeconomic disadvantage (Schhneider et al., 2006). In our study sample of UN-Spanish Hispanics, lower education and income were accompanied by low access to health insurance. Un- or under-insurance was a significant predictor of FT, especially coping FT, in our analysis. Once accounting for un- and under-insurance, we explored for an independent effect of language acculturation on FT but did not identify statistically significant effects in comparisons of FT for high vs. low English proficiency Hispanics. Possibly the qualitatively reported mitigating support resources such as church or extended family may offer critical protective effects on FT in this population with language barriers, potentially consistent with the mitigating effect of social support on FT identified in a recent qualitative study of Hispanic breast cancer patients (Chebli et al., 2020). Notably, while respondents in our study also qualitatively identified the financial assistance provided through the county public health program as another mitigating factor, respondents did not identify use of a wider spectrum of potential formal assistance resources outside the healthcare system, such as charity and other professional organizations. As the need for access to variety of formal assistance resources to help mitigate FT was identified in another qualitative study of diverse breast cancer patients with financial barriers (Gharzai et al., 2021), future studies underlying the barriers to knowledge and use of community assistance resources for cancer-related FT in low English proficiency Hispanics may elicit key intervenable factors (social determinants factors) and needs (the spectrum of social needs as well as needs specifically related to FT) in this population.

Notably, this pilot sample size was limited, and therefore the exploratory model in the present analysis is not sufficient to rule out independent effects of language acculturation on FT. Further, what is likely is that the language acculturation aspect interacts in complex ways with the socioeconomic, neighborhood, and healthcare environment factors before, during, and after diagnosis and treatment to influence FT outcomes. Our practical Spanish language FT measurement items are applicable to support additional investigations of this question.

This study identified distinct characteristics of FT among underserved, low English language proficiency Hispanic patients to guide and incorporate into future investigations. UN-Spanish Hispanic patients frequently identified a complete lack of resources such as income, savings, or credit cards and severe basic needs privations. Studies of FT with a focus on loss or decline of wealth (e.g., worsened credit score, defaulted mortgages, or loss of savings, retirement, or assets) (Katz et al., 2017), or measures such as the Comprehensive Score for Financial Toxicity (COST) (de Souza et al., 2017), that do not include specific basic needs privation could lead to gaps in representation of the severity and dimensions of FT in populations such as this. To continue adding to the available scientific evidence, these aspects of FT need to be represented in future studies of medically underserved Hispanic cancer patients (Ashing-Giwa et al., 2004; Blinder et al., 2012; Jagsi et al., 2014; Lee and Salloum, 2016; Jagsi et al., 2018; Shankaran et al., 2022). Providing options in responses to indicate that credit, savings, or income may not be relevant due to a complete lack of resources is important to avoid inaccurate floor effects in scoring. Multi-dimensional FT assessment, such as that provided by the ENRICh instrument, is needed to discern the aspects of material burden, coping, and psychological impact of FT. Prior large population studies of US Hispanics demonstrate the conflicting evidence on the severity and spectrum of FT (Ashing-Giwa et al., 2006; Panzone et al., 2021), potentially due to the heterogeneity of aspects of FT. Our results may help bridge the conflicting evidence, with our analysis demonstrating a wide variation in FT within this entirely Hispanic sample, for example, between the insured English-speaking vs. uninsured low English proficiency subgroups—variation that may be diluted in analyses of Hispanics as a single category.

This study has limitations to consider. There was a limited sample size and statistical power, and therefore the models analyzing the correlates of overall and subdomain FT are exploratory. The comparison groups were obtained as a convenience sample at only two institutions. In addition, the UN-English interviews were conducted at the onset of the COVID-19 pandemic whereas the INS-English surveys were conducted years prior to the pandemic, and therefore the potential economic factors that were impacting the participants enrolled before vs. after the pandemic may have differed. Furthermore, the comparisons between the groups in the analysis are still exploratory and require additional validation as well as examination of multi-level contributions to variation in outcome (i.e., organization-levels vs. patient-level effects). A strength of the study samples was the high density of Hispanics in the Houston metropolitan area from which they were drawn, which includes populations of mostly Mexican (76%), Salvadoran (8%), and Honduran (3%) origin (Demographic and Economic Profiles of Hispanics by State and County, 2011; Hispanic Population and Origin in Select U.S, 2016), reflecting the diversity of the US national Hispanic population (Key facts about U.S., 2019). However, future studies are still needed to fully understand the impact of neighborhood, geographic, ethnic, immigration status, and generational factors, given the heterogeneity of the US Hispanic population with even more broadened inclusion.

## 5. Conclusion

In this pilot study focused on un- and under-insured Hispanics with cancer, comprehensive FT measurement with the ENRICh FT measure in high-risk, low English proficiency individuals was feasible, acceptable, and internally consistent when administered in Spanish. While the results provide a tool for practical and useful Spanish language measurement items to assess multi-dimensional FT in additional research and practice, they also emphasize that future studies employing FT assessments focused on high-risk Hispanic populations need to encompass the types of privations and economic hardships this population uniquely faces, such as severe basic needs privations and deficient or lacking access to resources such as savings and credit. The results of this study identified that inadequate insurance was a potential predictor of FT among Spanish- and English-speaking Hispanics. While language acculturation was not found to be an independent risk for FT in exploratory analysis, further exploration of the differences among lower and higher English proficiency in additional diverse subpopulations continue to be warranted, especially to disentangle the potential effects of language proficiency, sociodemographic factors, and insurance factors. The initial findings from this pilot study provide practical insights and items for FT assessment in future practice and research as well as key understandings for tailoring ongoing efforts in financial toxicity measurement, assessment, and intervention to meet the specific needs of underserved Hispanic populations with low English language proficiency.

## Data availability statement

The original contributions presented in the study are included in the article/Supplementary material, further inquiries can be directed to the corresponding author.

## Ethics statement

The studies involving human participants were reviewed and approved by University of Texas M. D. Anderson Cancer Center Institutional Review Board. The patients/participants provided their written informed consent to participate in this study.

## Author contributions

SP, TM, SS, RV, SG, and GS: contributed significantly to the experimental design. GM, SP, Y-SC, KK, NT, HM, and D-KN: implementation. JS, GM, SP, RY, C-FW, SS, Y-CS, and GS: analysis and interpretation of the data. All authors contributed to the article and approved the submitted version.

## Funding

GS was supported by the National Cancer Institute (NIH/NCI K07CA211804) for components of this research. This research was supported in part by the MD Anderson Cancer Center grant P30 CA016672 (C-FW) and the Decision Science Shared Resource (RV). SG receives research funding support from CPRIT RP160674, CPRIT RP210140, and Komen SAC150061 not directly related to this study. This work was supported in part by the Boone Pickens Distinguished Chair for Early Prevention of Cancer to E. Hawk and The University of Texas MD Anderson Cancer Center Duncan Family Institute for Cancer Prevention and Risk Assessment and the NIH/NCI under award number P30CA016672.

## Conflict of interest

The authors declare that the research was conducted in the absence of any commercial or financial relationships that could be construed as a potential conflict of interest.

## Publisher’s note

All claims expressed in this article are solely those of the authors and do not necessarily represent those of their affiliated organizations, or those of the publisher, the editors and the reviewers. Any product that may be evaluated in this article, or claim that may be made by its manufacturer, is not guaranteed or endorsed by the publisher.
