# Supplementary material for: Pilot study of a Spanish language measure of financial toxicity in underserved Hispanic cancer patients with low English proficiency
Source: Front Psychol. 2023 Jul 10;14:1188783. doi: 10.3389/fpsyg.2023.1188783 (PMC10364629; doi:10.3389/fpsyg.2023.1188783)
Supplement: Supplementary file 1 [file Data_Sheet_1.docx]

Supplementary Material

**Pilot Study of a Spanish Language Measure of Financial Toxicity in Underserved Hispanic Cancer Patients with Low English Proficiency**

**Supplementary Material Table 1.** Financial Toxicity Instrument Items: Spanish Translation

| **Item (English)** | **Spanish Translation** |
| --- | --- |
| ***Rated for the past month, respondents score severity of disease or treatment impact on the item or reliance on the resource to cope with financial impact related to disease or treatment.*** | |
| **Material financial toxicity** |  |
| Money in savings | El dinero en sus ahorros |
| Debts or other money owed | Otro dinero que posee usted (ej., deudas y tarjetas de crédito) |
| Spending on medical bills | Sus gastos en facturas médicas |
| Using household income | Uso de sus ingresos del hogar |
| Using savings | Uso de sus ahorros |
| Using credit cards | Uso de tarjetas de crédito |
| **Coping financial toxicity** |  |
| Ability to pay all bills | Su capacidad para pagar todas sus facturas |
| Ability to pay for food | Su capacidad para pagar la comida |
| Ability to work usual number of hours at job | Su capacidad para trabajar el número de horas habitual es en su empleo |
| Ability to contribute to normal household responsibilities | Su capacidad para contribuir a sus responsabilidades domésticas normales y tareas diarias |
| Assistance with managing medical bills | Contar con alguien que le ayudara a administrar sus facturas médicas |
| Assistance with typical responsibilities | Contar con alguien que le ayudara con sus responsabilidades domésticas normales y tareas diarias |
| Assistance with care for dependents | Contar con alguien que le ayudara a cuidar a quienes normalmente dependen de usted |
| Assistance from community | Contar con ayuda de recursos de la comunidad (como iglesias, fundaciones, asistencia a pacientes, etc.) |
| **Psychological financial toxicity** |  |
| Stress level about finances | Su nivel de estrés en cuanto a las finanzas |

**Supplementary Material Table 2.** Univariate Correlates of Severe Overall Financial Toxicity (score >6)

|  | |  |  |
| --- | --- | --- | --- |
| **Patient Characteristic** | **Severe FT**  **N of 27 (%)** | **P-value** |  |
|  |  |  |  |
|  |  |  |  |
| Age, Mean (SD) | 47.9(15.8) | 0.20 |  |
| Gender % (n) |  | 0.087 |  |
| Male | 13(48.2) |  |  |
| Female | 14(51.9) |  |  |
| Neighborhood Area Deprivation Index Score, National Percentile, Mean (SD) | 64.1(21.6) | 0.86 |  |
| Currently Working For Pay |  | 0.11 |  |
| Yes | 4(14.8) |  |  |
| No | 23(85.2) |  |  |
| Income |  | 0.057 |  |
| <$10,000 | 9(33.3) |  |  |
| $10,000-$34,999 | 8(29.6) |  |  |
| $35,000-$49,999 | 2(7.4) |  |  |
| $50,000-$99,999 | 2(7.4) |  |  |
| >$100,000 | 0(0) |  |  |
| No response | 6(22.2) |  |  |
| Education |  | 0.29 |  |
| Less than High School, High School, or GED | 16(59.3) |  |  |
| Some College, Associate Degree, or Trade Certification | 6(22.2) |  |  |
| College, Graduate, or Advanced Degree | 1(3.7) |  |  |
| No Response | 4(14.8) |  |  |
| Marital Status |  | 0.34 |  |
| Married/Living as Married | 11(40.7) |  |  |
| Other | 16(59.3) |  |  |
| Insurance |  | **0.028** |  |
| Insurance Provided Through Employer or Purchased | 5(18.5) |  |  |
| Medicaid or Other State Provided Insurance | 2(7.4) |  |  |
| Medicare | 2(7.4) |  |  |
| No Insurance | 18(66.7) |  |  |
| Received Chemotherapy |  | **0.006** |  |
| Yes | 27(100) |  |  |
| No | 0(0) |  |  |
| Advanced or Metastatic Cancer at Diagnosis |  | 0.075 |  |
| Yes | 22(81.5) |  |  |
| No | 5(18.5) |  |  |
